# Effects of modified electroconvulsive therapy on the electroencephalogram of schizophrenia patients

**DOI:** 10.1186/s40064-016-2747-7

**Published:** 2016-07-12

**Authors:** Ling Zhao, Yansheng Jiang, Hongxing Zhang

**Affiliations:** The Second Affiliated Hospital of Xinxiang Medical University, No. 388 Middle Jianshe Road, Xinxiang, 453002 Henan China; The Psychology Department of Xinxiang Medical University, Xinxiang, 453003 Henan China

**Keywords:** Schizophrenia, Modified electroconvulsive therapy, Electroencephalogram

## Abstract

**Background:**

This study aimed to investigate the modified electroconvulsive therapy (MECT) on the electroencephalogram (EEG) of schizophrenia patients. A total of 26 schizophrenia patients who received MECT were recruited. EEG recording was initiated at 30 min before 1st and 6th MECT and terminated on the 2nd day. Images without artifacts were selected for the analysis of δ, θ, α1, α2 and β bands. The wave energy at each frequency, index of waves at different bands from the same lead, index of waves at the same band from different leads, time of epileptic discharge, time of resting state, and time to the stable EEG were determined and compared.

**Results:**

The energy of slow waves increased. α waves reduced, but θ waves increased in the frontotemporal area. The index of θ waves increased. After resting state, brainwaves first occurred in the frontal area. Significant difference was observed in the time to waves returning to normal (*P* = 0.05).

**Conclusions:**

After MECT, the θ waves in the same lead increases, and its energy also elevates; α wave in the frontotemporal area reduces; there is transient reduction in cerebral function during MECT. After electric resting state, brainwaves mainly occur in the frontal area, and the time to brainwaves returning to normal reduces over time after MECT.

## Background

Schizophrenia (SZ) is a group of mental disorders of unknown causes. Clinically, SZ patients present false beliefs, thought disorder, auditory hallucinations, reduced social engagement and emotional expression, and lack of motivation. Generally, SZ patients have conscious awareness and normal intelligent, but a few patients may develop cognition impairment during the disease progression. SZ is a mental disease with high morbidity, high recurrence rate and high disability. SZ has been regarded a mental disease with the leading burden in twentyfirst century in the China workshop on burden of disease (The Ministry of Health, The Ministry of Civil Affair and China Disabled Persons’ Federation 2003).

Currently, SZ is regarded as a disease with brain function disorder (Yang and Wang 2001; Sun 2006; Rogasch et al. 2014), and electroencephalography (EEG) is a common strategy for the examination of cerebral function. EEG is the graphical representation of voltage fluctuations recorded through the scalp or intracranially that reflect the electrical activity of large assemblies of neurons. EEG can be used to evaluate the extent and severity of brain injury and provide information for the evaluation of prognosis.

The therapy of SZ has been a focus in the psychiatric studies. Electroconvulsive therapy (ECT) is a physical therapy in the Department of Psychiatry in our hospital in China, in which seizures are electrically induced in patients to provide relief from psychiatric illnesses. The mechanism underlying the therapeutic effect of ECT is still unclear (Chen et al. 2009), but it has definite efficacy in SZ and depression (Wang et al. 2015; Rybakowski et al. 2016). Modern ECT is still based on the induction of a brief seizure in a highly controlled setting, while the motor signs of the seizure are absent. Compared with traditional ECT, MECT is safer, and may achieve effectiveness sooner and has definite efficacy. Generally, MECT-induced seizure is closely related to the therapeutic effect of MECT. Of note, intravenous anesthetics and muscle relaxant are used during MECT and thus seizure may not be observed, but seizure-related waves can be found in the EEG. The change in cerebral function demonstrated by EEG might indicate the mechanism underlying the therapeutic effect of MECT.

In the present study, SZ patients receiving MECT were recruited. EEG was recorded at 1st and 6th MECT and compared, which may provide information on the change in cerebral function following MECT. This study aimed to investigate the MECT on the EEG of schizophrenia patients.

## Methods

### Patients

This study was approved by the Ethics Committee of the Second Affiliated Hospital of Xinxiang Medical University, and complying with the Helsinki Declaration. Patients who were hospitalized in the Second Affiliated Hospital of Xinxiang Medical University were recruited between February 2012 and March 2013. These patients were diagnosed with SZ according to the DSM-IV diagnostic criteria for SZ. Informed consent was obtained from each patient or their guardians, and MECT did not interfere with the pre-existing therapy. All the patients were maintained with clozpine before and after MECT. A total of 26 patients were included in this study. There were 12 males and 14 females with the mean age of 26.3 ± 6.2 years old (range 18–42). The mean course of disease was 2.2 ± 1.9 years (range 6 months–8 years). According to physicians’ judgment, these patients were included in accordance with indications of MECT. And they had no history of vaccination or infection recently. Comparisons were conducted between data collected before and after therapy. During treatment, presence of allergy and other events were indicative of withdrawal from this study.

### MECT

MECT was conducted according to the standard protocol in the instructions of MECT therapeutic apparatus thrice weekly for 2 weeks. Chloride succinylcholinet (0.6–1.0 mg/kg) was applied as muscle relaxant, atropine sulfate (0.5–1.0 mg) was used to induce anesthesia, and propofol (1.5–2 mg/kg) was used to maintain anesthesia. The antipsychotic therapy remained stable during MECT except for the discontinuation of pharmacotherapy in the morning on the day of MECT. Routine nursing was administered, and patients received food and water deprivation for 6 h before MECT. Intravenous access was prepared.

Multifunction electric shock therapy instrument (SOMATIC, USA) was used for MECT at DGX mode. The charge was adjusted according to patients’ age. This instrument can also be used to monitor the EEG and ECG. The static resistance was 100–3000 Ω, and electrodes were placed at bilateral temporal lobes.

### EEG recording

Ag–AgCl electrodes were placed according to the international 10–20 system, including a total of 8 recording electrodes (FPl, FP2, C3, C4, O1, O2, T3 and T4), an indifferent electrode (FZ) and 2 ear electrodes (A1 and A2). The scalp was cleared with ethanol and the electrodes were fixed on the scalp with collodion. Both ear electrodes served as reference electrodes (Ref) for single-lead recording. The sensitivity was 100 μV (1 cm), the high-frequency filter (HF) was 30 Hz, the time constant (TC) was 0.3 s, notch filter was 50 Hz, and the scalp impedance of each electrode was no higher than 5000 Ω.

After recording, the EEG was subjected to digital analysis. The energy of waves at each band, index (distribution) of waves at different bands from the same lead, and index of waves at the same band in different leads were determined. The frequency of δ, θ, α1, α2 and β waves was 0.5–3, 3–8, 8–10, 10–13 and 12–30 Hz, respectively. The time of epileptic discharge, time of electric resting state, and time to stable EEG were also recorded.

### Statistical analysis

Statistical analysis was performed with SPSS version 17.0. Comparisons between two groups were conducted with paired t test. A value of *P* < 0.05 was considered statistically significant.

## Results

Results of comparison of PANSS scores before MECT and after six times of treatments indicated that significant difference was observed in PANSS score before and after MECT (*P* < 0.05; Table 1).

**Table 1:** PANSS score before and after MECT

	n	$\bar{x} \pm s$	*t*	*p*
Before treatment	26	93.75 ± 25.60	4.891	0.000
After treatment	26	57.00 ± 1975		

Energy of waves at different frequencies at the 1st MECT and 6th MECT in SZ patients are shown in Table 2. Significant difference was observed in the energy of wave at different bands detected at the 1st MECT and 6th MECT (δ, θ, α2 and β in FP1 lead; θ, α1 and β in FP2 lead; θ, α1, α2 and β in C3 lead; θ, α1, α2 and β in C4 lead; δ, θ, α2 and β in O1 lead; δ, θ, α2 and β in O2 lead; β in T3 lead; α1 in T4 lead; *P* < 0.05). When compared with energy of waves at the 1st MECT and 6th MECT, the energy of slow waves (δ and θ) increased (Table 2).

**Table 2:** Energy of waves at different frequencies at the 1st MECT and 6th MECT in SZ patients

Electrode	Bands	1st (n = 26)	6th (n = 26)	*t*	*P*
FP1	δ	60.00 ± 19.83	77.32 ± 32.35**	−3.216	0.005
	θ	70.08 ± 22.74	116.19 ± 59.92*	−4.374	0.022
	α1	32.73 ± 11.63	34.51 ± 12.35	−0.624	0.187
	α2	31.33 ± 3.76	33.52 ± 8.44**	−1.737	0.000
	β	14.73 ± 3.25	15.61 ± 4.42**	−1.320	0.000
FP2	δ	62.20 ± 16.37	97.09 ± 51.70	−3.695	0.064
	θ	74.55 ± 24.39	113.53 ± 65.84**	−4.963	0.008
	α1	37.01 ± 14.72	38.53 ± 12.48**	−0.658	0.000
	α2	32.17 ± 6.04	37.03 ± 10.85	−2.237	0.239
	β	15.12 ± 3.46	17.25 ± 4.89**	−3.391	0.000
C3	δ	44.90 ± 6.65	60.92 ± 38.67	−2.186	0.167
	θ	67.22 ± 26.16	102.24 ± 52.15**	−4.130	0.003
	α1	33.41 ± 11.89	34.74 ± 10.32*	−0.568	0.029
	α2	37.04 ± 13.86	36.20 ± 14.10**	0.550	0.000
	β	14.52 ± 3.69	16.02 ± 7.59**	−1.303	0.000
C4	δ	47.85 ± 7.29	67.64 ± 38.23	−2.575	0.858
	θ	68.01 ± 33.42	113.12 ± 62.74**	−4.448	0.002
	α1	36.60 ± 14.20	39.14 ± 12.21**	−1.158	0.000
	α2	38.11 ± 10.93	40.33 ± 14.34**	−0.932	0.007
	β	15.76 ± 4.04	18.24 ± 7.87**	−2.030	0.001
O1	δ	54.55 ± 17.52	67.54 ± 42.99**	−2.120	0.000
	θ	72.95 ± 31.95	113.44 ± 74.62***	−3.209	0.007
	α1	44.28 ± 21.38	47.93 ± 17.12	−0.792	0.177
	α2	49.14 ± 16.69	53.05 ± 22.17**	−1.293	0.000
	β	17.87 ± 5.25	18.93 ± 6.81*	−0.859	0.013
O2	δ	55.75 ± 16.43	82.92 ± 58.02**	−2.973	0.000
	θ	81.43 ± 31.50	131.13 ± 88.83**	−3.363	0.004
	α1	50.63 ± 22.89	52.74 ± 13.43	−0.429	0.594
	α2	55.86 ± 22.47	56.49 ± 22.62*	−0.137	0.016
	β	19.12 ± 4.87	22.11 ± 9.27*	−1.878	0.012
T3	δ	35.48 ± 7.51	61.47 ± 55.44	−2.366	0.969
	θ	49.45 ± 18.46	85.53 ± 70.06	−2.629	0.507
	α1	22.15 ± 8.36	25.07 ± 8.89	−1.233	0.921
	α2	24.41 ± 5.69	26.23 ± 10.77	−0.877	0.134
	β	11.15 ± 2.92	12.30 ± 4.14	−1.421	0.077
T4	δ	50.18 ± 21.97	71.02 ± 46.26	−2.123	0.777
	θ	62.84 ± 23.36	101.67 ± 57.54	−3.623	0.107
	α1	28.45 ± 11.53	33.55 ± 9.34*	−2.402	0.014
	α2	29.11 ± 8.25	34.20 ± 9.23	−2.097	1.000
	β	15.08 ± 5.56	16.97 ± 5.82	−1.224	0.821

* *P* < 0.05; ** *P* < 0.01

Index of waves at different bands in the same lead at the 1st MECT and 6th MECT in SZ patients are shown in Table 2. There was significant difference in the index of waves at different bands in the same lead detected at the 1st MECT and 6th MECT (δ, α1, α2 and β in FP1 lead; δ, θ, α1, α2 and β in FP2 lead; θ, α1, α2 and β in C3 lead; δ, θ, α1 and β in C4 lead; θ, α1, α2 and β in O1 lead; δ, α1 and α2 in O2 lead; θ and β in T3 lead; and θ, α1 and β in T4 lead; *P* < 0.05; Table 3).

**Table 3:** Index of waves at different bands in the same lead at the 1st MECT and 6th MECT in SZ patients

Electrode	Band	1st (n = 26)	6th (n = 26)	*t*	*P*
FP1	δ	28.46 ± 6.10	28.37 ± 6.68**	−0.118	0.000
	θ	32.91 ± 5.10	34.98 ± 12.43	0.791	0.917
	α1	12.76 ± 4.89	12.80 ± 2.79**	−3.612	0.006
	α2	15.58 ± 3.39	12.15 ± 3.01*	−4.923	0.048
	β	7.30 ± 2.05	5.92 ± 1.67**	−6.083	0.000
FP2	δ	28.35 ± 5.54	29.95 ± 7.01**	1.434	0.001
	θ	33.18 ± 4.75	38.29 ± 7.59**	4.523	0.000
	α1	16.56 ± 4.97	12.69 ± 3.07**	−5.115	0.001
	α2	14.97 ± 2.66	12.50 ± 3.97**	−4.896	0.000
	β	7.00 ± 1.67	5.72 ± 1.75**	−4.720	0.000
C3	δ	23.58 ± 5.26	23.91 ± 4.93	0.293	0.067
	θ	33.28 ± 5.82	39.66 ± 5.10**	7.445	0.000
	α1	16.73 ± 4.15	14.69 ± 3.07**	−3.228	0.001
	α2	18.861 ± 5.80	15.21 ± 3.43*	−3.571	0.019
	β	7.50 ± 2.11	6.50 ± 1.64**	−2.925	0.001
C4	δ	23.66 ± 5.74	24.16 ± 4.26*	0.474	0.020
	θ	33.70 ± 5.45	39.27 ± 5.35**	6.050	0.001
	α1	16.89 ± 4.22	14.73 ± 3.19**	−3.564	0.000
	α2	17.64 ± 4.50	15.19 ± 3.04	−2.533	0.354
	β	7.63 ± 2.03	6.61 ± 1.48**	−3.726	0.000
O1	δ	23.18 ± 4.90	21.80 ± 4.34	−1.301	0.114
	θ	29.77 ± 5.20	35.53 ± 5.81**	5.591	0.004
	α1	18.13 ± 5.85	17.63 ± 3.80**	−0.724	0.000
	α2	21.03 ± 6.26	18.40 ± 4.72*	−2.170	0.049
	β	7.87 ± 2.54	6.61 ± 1.81**	−2.979	0.003
O2	δ	21.22 ± 5.49	23.35 ± 4.84*	1.966	0.026
	θ	29.16 ± 7.65	36.03 ± 5.91	4.486	0.072
	α1	18.32 ± 5.41	16.56 ± 4.25**	−2.350	0.000
	α2	20.93 ± 6.13	17.31 ± 4.51*	−3.348	0.010
	β	8.16 ± 3.01	6.73 ± 1.11	−2.423	0.156
T3	δ	25.33 ± 5.62	28.184 ± 5.84	1.909	0.572
	θ	33.81 ± 5.95	37.72 ± 6.22**	3.600	0.002
	α1	15.33 ± 3.72	13.33 ± 3.01*	−2.810	0.026
	α2	17.47 ± 4.06	14.05 ± 3.54	−4.085	0.057
	β	8.04 ± 2.36	6.70 ± 1.90**	−3.177	0.008
T4	δ	26.98 ± 6.96	26.92 ± 5.28	−0.042	0.173
	θ	33.20 ± 5.42	38.10 ± 5.41*	4.359	0.024
	α1	15.15 ± 4.29	13.75 ± 2.74*	−1.807	0.025
	α2	16.14 ± 3.93	14.28 ± 3.48	−2.246	0.076
	β	8.50 ± 3.24	6.95 ± 1.80****	−2.901	0.004

* *P* < 0.05; ** *P* < 0.01

Index of waves at the same band in different leads at the 1st MECT and 6th MECT in SZ patients are shown in Table 3. There was significant difference in the index of waves at the same band in different leads detected at the 1st MECT and 6th MECT (δ, θ, α2 and β in FP1 lead; α2 and β in FP2 lead; δ, θ, α1, α2 and β in C3 lead; α1, α2 and β in C4 lead; δ, α1 and α2 in O1 lead; α1 and α2 in O2 lead; and θ in T3 lead; *P* < 0.05; Table 4).

**Table 4:** Index of waves at the same band in different leads at the 1st MECT and 6th MECT in SZ patients

Electrodes	Bands	1st (n = 26)	6th (n = 26)	*t*	*P*
FP1	δ	14.38 ± 2.53	13.99 ± 3.42**	−0.927	0.000
	θ	12.92 ± 2.38	13.22 ± 1.77**	0.822	0.000
	α1	11.70 ± 1.89	11.12 ± 2.18	−1.182	0.202
	α2	10.98 ± 2.43	10.80 ± 1.74**	−0.494	0.000
	β	12.17 ± 2.91	11.58 ± 1.75**	−1.341	0.000
FP2	δ	15.06 ± 2.03	16.83 ± 3.26	2.611	0.273
	θ	13.73 ± 2.13	14.89 ± 2.00	2.572	0.061
	α1	13.06 ± 2.40	12.50 ± 2.03	−1.127	0.076
	α2	11.30 ± 3.11	11.88 ± 2.22**	1.184	0.001
	β	12.45 ± 2.89	12.86 ± 2.05**	0.853	0.003
C3	δ	11.08 ± 1.23	10.35 ± 1.79**	−2.630	0.001
	θ	12.14 ± 1.15	11.69 ± 2.09**	−1.426	0.000
	α1	11.83 ± 1.53	11.33 ± 2.78**	−1.660	0.000
	α2	12.29 ± 2.38	11.43 ± 2.41**	−2.051	0.001
	β	11.43 ± 1.32	11.33 ± 2.07**	−0.330	0.000
C4	δ	11.90 ± 1.38	11.65 ± 1.02*	−0.930	0.039
	θ	13.15 ± 1.30	12.83 ± 1.06	−1.137	0.188
	α1	13.46 ± 3.06	12.56 ± 1.67**	−1.804	0.002
	α2	13.09 ± 2.29	12.68 ± 1.36**	−1.346	0.000
	β	12.16 ± 2.11	12.94 ± 1.73*	2.046	0.010
O1	δ	13.36 ± 3.38	11.44 ± 2.07*	−2.633	0.021
	θ	13.03 ± 1.89	12.55 ± 1.68	−0.874	0.177
	α1	15.13 ± 2.69	16.36 ± 3.15**	2.137	0.008
	α2	16.24 ± 2.44	16.49 ± 2.85*	0.246	0.048
	β	14.35 ± 2.25	13.80 ± 1.83	−0.963	0.937
O2	δ	13.53 ± 2.20	13.86 ± 2.28	0.604	0.199
	θ	14.66 ± 1.86	14.43 ± 2.07	−0.462	0.271
	α1	17.66 ± 4.58	17.20 ± 2.77*	−0.581	0.018
	α2	18.34 ± 3.98	17.60 ± 3.23**	−1.039	0.007
	β	15.46 ± 2.13	15.99 ± 2.79	0.762	0.922
T3	δ	8.72 ± 1.52	9.83 ± 2.49	1.882	0.717
	θ	8.97 ± 1.21	8.85 ± 1.93*	−0.220	0.032
	α1	7.92 ± 1.41	8.04 ± 1.43	0.284	0.744
	α2	8.31 ± 1.41	8.23 ± 1.48	−0.179	0.457
	β	9.04 ± 1.50	9.10 ± 1.60	0.150	0.680
T4	δ	12.01 ± 3.56	12.05 ± 1.49	0.045	0.072
	θ	11.45 ± 2.57	11.52 ± 1.22	0.117	0.439
	α1	9.90 ± 1.68	10.85 ± 1.22	2.526	0.468
	α2	9.81 ± 1.73	10.88 ± 1.29	2.987	0.151
	β	12.26 ± 3.74	12.38 ± 1.02	0.159	0.373

* *P* < 0.05; ** *P* < 0.01

As shown in Tables 3 and 4, when compared with EEG at the 1st MECT, the number of α wave in the frontal areas (frontotemporal area) reduced, but that of θ wave increased at the 6th MECT. In addition, the index of α wave reduced in all the leads, but that of θ wave increased.

### Evolution of EEG

Of 26 patients, all phenomenons such as the epileptic discharge, electric resting state and waves returning to normal were observed in 25 patients, and the electric resting state was not found in 1 patient.

**Fig. 1:**
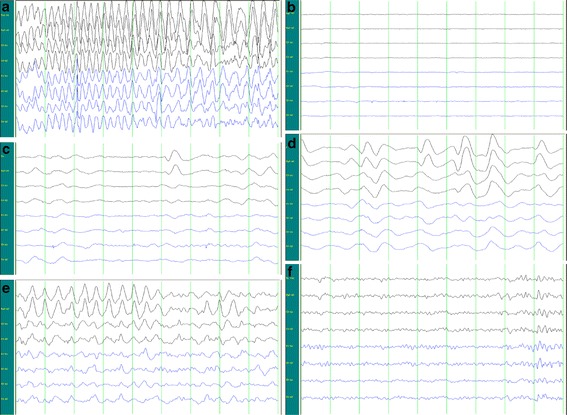
**a** Epileptic discharge when the current was switched on; **b** EEG at resting state after epileptic discharge; **c** After resting stage, brainwaves occurred first in the frontal lobe, their amplitudes were low and their frequencies were slow. **d** In the progression of brainwaves, the frequencies became slow, but these waves were slow waves that were mainly found in the δ band. **e** The amplitude reduced, but frequency increased gradually. In addition, brainwaves at δ and θ bands were found, but only a few brainwaves were noted at α band. **f** Amplitude further reduced and frequency further increased, and then brainwaves retuned to stable level

After electric resting state, the brainwaves returned to stable and normal. The brainwaves showed the dynamic change over time. Brainwaves were first found in the frontal region, or occurred in all the leads but their amplitudes were the highest in the frontal lobe. Their amplitude then decreased and frequencies increased gradually over time. Figure 1 showed the progression of brainwaves in the same patient.

### Time of epileptic discharge, time of electric resting state and time to EEG normalization

The time of epileptic discharge, time of electric resting state and time to EEG normalization were determined at the 1st MECT and 6th MECT and compared (Table 5).

**Table 5:** Time of epileptic discharge, time of electric resting state and time to EEG normalization at the 1st MECT and 6th MECT

	1st MECT	6th MECT	*t*	*P*
Time of epileptic discharge	45.28 ± 40.93	38.92 ± 15.54	0.753	0.459
Time of electric resting state	25.28 ± 14.69	21.30 ± 11.99	1.030	0.313
Time to brainwaves returning to stable	724.56 ± 290.03	600.96 ± 263.44**	3.126	0.005

** *P* < 0.01

There were no significant differences in the time of epileptic discharge and time of electric resting state, but marked difference was observed in the time to EEG normalization. This suggests that, with the increase in MECT, the time to returning to regular discharge for brain cells reduces after short resting state.

## Discussion

SZ has various manifestations and its pathogenesis is still unclear. Generally, SZ is regarded a disease of brain dysfunction (Yang and Wang 2001; Sun 2006; Rogasch et al. 2014). Therapy of SZ has been a focus in psychiatric studies. Electroconvulsive therapy is a common physical therapy in the department of psychiatric diseases (Rey and Walter 1997), but the mechanism underlying the therapeutic effect of electric shock therapy is still unclear although it has definite therapeutic efficacy (Danese 2006). A majority of investigators have investigated the brain dysfunction secondary to ECT or MECT, but studies are mainly confined to imaging examinations and psychological examinations (Lerer et al. 1995; Wengel et al. 1998; Barnes et al. 1997). The brain function is mainly evaluated by EEG. However, few studies have been conducted to investigate the effects of MECT on EEG. In the present study, the EEG was analyzed after MECT in SZ patients.

EEG is the summary of electrical activities of regional neurons that are recorded through the scalp or intracranial electrodes. EEG reflects the net consequence of postsynaptic potentials of numerous neurons recorded by electrodes. Internationally, the brainwaves are classified as δ, θ, α and β according to the bands, the frequency range of which is 0.3–3, 4–7, 8–13 and 14–30 Hz, respectively. Brainwaves of 30 Hz or higher are also known as γ waves. Abnormalities in EEG have been confirmed in SZ patients. Chen et al. (Chen and Liu 2010) found that slow waves (δ and θ waves, especially θ wave) increased significantly, and α wave in the frontal lobe increased. There is evidence showing that the abnormalities in EEG of SZ patients are characterized by changes in rapid waves (Bandyopadhyaya et al. 2011). In addition, studies also indicate that changes in rapid waves are major abnormalities in SZ patients with a short course of disease, but both rapid waves and θ and α waves also show abnormalities in SZ patients with a long course of disease. The EEG is more complex in SZ patients than in healthy subjects, which also suggests the difference in EEG between healthy controls and SZ patients. These findings are mainly from studies that compare SZ patients with healthy controls. In this study, SZ patients were recruited, and EEG was compared at the 1st MECT and 6th MECT. Our results showed the index of α wave in each lead reduced, especially in the frontotemporal area with the increase in MECT, but the energy and index of θ wave increased, suggesting the brain dysfunction. These results were consistent with previous findings (Chen and Liu 2010; Bandyopadhyaya et al. 2011; von Stein and Sarnthein 2000; Boutros et al. 2008; Breakspear 2006; Hornero et al. 2006; Cheng and Shen 2012) and also reflected the therapeutic effects of MECT.

In the present study, the EEG was investigated in 26 SZ patients. Of these patients, epileptic discharge, electric resting state and EEG normalization were observed in 25 patients, and electric resting state was not found in 1 patient. After electric resting state, the brainwaves returned to normal level. Initially, brainwaves were characterized by large slow waves, which was even lower than 0.5 Hz. Then, the frequency increased gradually, the amplitude increased first, then reduced and became stable finally, which were consistent with the dynamic change in brainwaves over age (Huang and Wu 1984; Liu 2006b). The change in brainwaves following MECT may be ascribed to the reconstruction of cerebral function, which improves the discharge of original neurons or the nerve conduction loop, exerting therapeutic effect.

Currently, it has been proposed that the electric activities of the neurons recorded through the cortex or scalp are mainly the postsynaptic potentials of apical dendrites of pyramidal cells in the cortex. The number of neurons generating synchronous activities, voltage and frequency are important factors determining the electric activities recorded through the scalp. The EEG rhythms originate from the synchronised activity of cortical pyramidal cells and then spread and expand. The brainwaves at different bands are related to the distribution of different neuronal nuclei (Liu 2006b). The large pyramidal cells are closely associated with the epileptic discharge. In the available theories, epileptic discharge originates from the limbic system, especially the hippocampus, and hippocampal loop and thalamus-cortex loop make the epileptic discharge become rhythmic and maintain this rhythm (Liu 2006a). Studies have indicated that brain-derived neurotrophic factor (BDNF) is widely expressed in the central nervous system and is crucial for the survival of neurons, functional maintenance of neurons and synaptic plasticity (Zarate et al. 2006). Animal experiments confirm that electric seizure may significantly increase BDNF in different areas of the brain (Burnouf et al. 2013; Hasselbalch et al. 2012; Gedge et al. 2012), indicating MECT may stimulate the expression of neurotrophic factors. However, the findings in available studies are conflicting (Gyekis et al. 2013). After epileptic discharge, electric resting state is present, followed by the activities of brainwaves, suggesting there is transient functional loss of neurons in the cortex, followed by the progressive recovery, and this is similar to the change in brainwaves during the development over age. We speculated that this reflected the rapid construction of brainwaves. There is evidence showing that MECT may alter the activities of biological rhythm center and the distribution of monoamine neurotransmitters to forcibly correct the function of the brain with existing dysfunction. In this study, electric resting state was not found in 1 patient before both MECT, which is required to be further investigated in our future studies with larger sample size.

Our results showed that time of epileptic discharge and time of electric resting state were comparable, but significant difference was found in the time to EEG normalization. This suggests that the time of epileptic discharge and time of electric resting state remain stable after MECT, but the time to EEG normalization is shortened with the increase in MECT, indicating the rapid recovery of cerebral function after MECT induced transient loss of cerebral function.

In the present study, brainwaves were first present in the frontal area or occurred in all the leads, but the brainwaves in the frontal area had the highest amplitude. The amplitude reduced and frequency increased gradually over time, which were different from the EEG of pediatric patients. The dominant brainwaves in pediatrics have not been found in the frontal area, but they first occur in the top central area and then spread to the occipital area (Rey and Walter 1997). Thus, we speculated that the brainwaves first occurred in the frontal area after electric resting state. We assumed that the presence of brainwaves after MECT might be similar to the dominant pacemaker in the electrocardiogram, but it required to be further studied. MECT may induce the super-synchronization of brain cells, resulting in epileptic discharge. After electric resting state, the discharge rhythm of neuronal nucli appears again, but the specific mechanism should be further studied. Our future studies on the neurotransmitters and imaging may provide more information.

There are still limitations in this study. The recruitment of SZ is relatively difficult, and the requirements for recording EEG are also high. Thus, the sample size is small in this study. This issue will be further investigated in our future studies with larger sample size.

## Conclusions

After MECT, the θ waves in the same lead increases, and its energy also elevates; α wave in the frontotemporal area reduces; there is transient reduction in cerebral function during MECT. After postictal suppression (voltage = 0; Fig. 1b), brainwaves (δ, θ, α1, α2 and β) mainly occur in the frontal area, and the time to brainwaves returning to normal (before MECT) reduces over time after MECT.

## Abbreviations

SZ: schizophrenia
EEG: electroencephalography
ECT: electroconvulsive therapy
MECT: modified electroconvulsive therapy
Ref: reference electrodes
HF: high-frequency filter
TC: time constant
BDNF: brain-derived neurotrophic factor
